# Supplementary material for: Dosage Compensation of the X Chromosome during Sheep Testis Development Revealed by Single-Cell RNA Sequencing
Source: Animals (Basel). 2022 Aug 24;12(17):2169. doi: 10.3390/ani12172169 (PMC9454834; doi:10.3390/ani12172169)
Supplement: Supplementary file 1 [file animals-12-02169-s001.zip › animals-1804171-supplementary.pdf]

Supplementary Materials: **Including 1 supplementary figure**

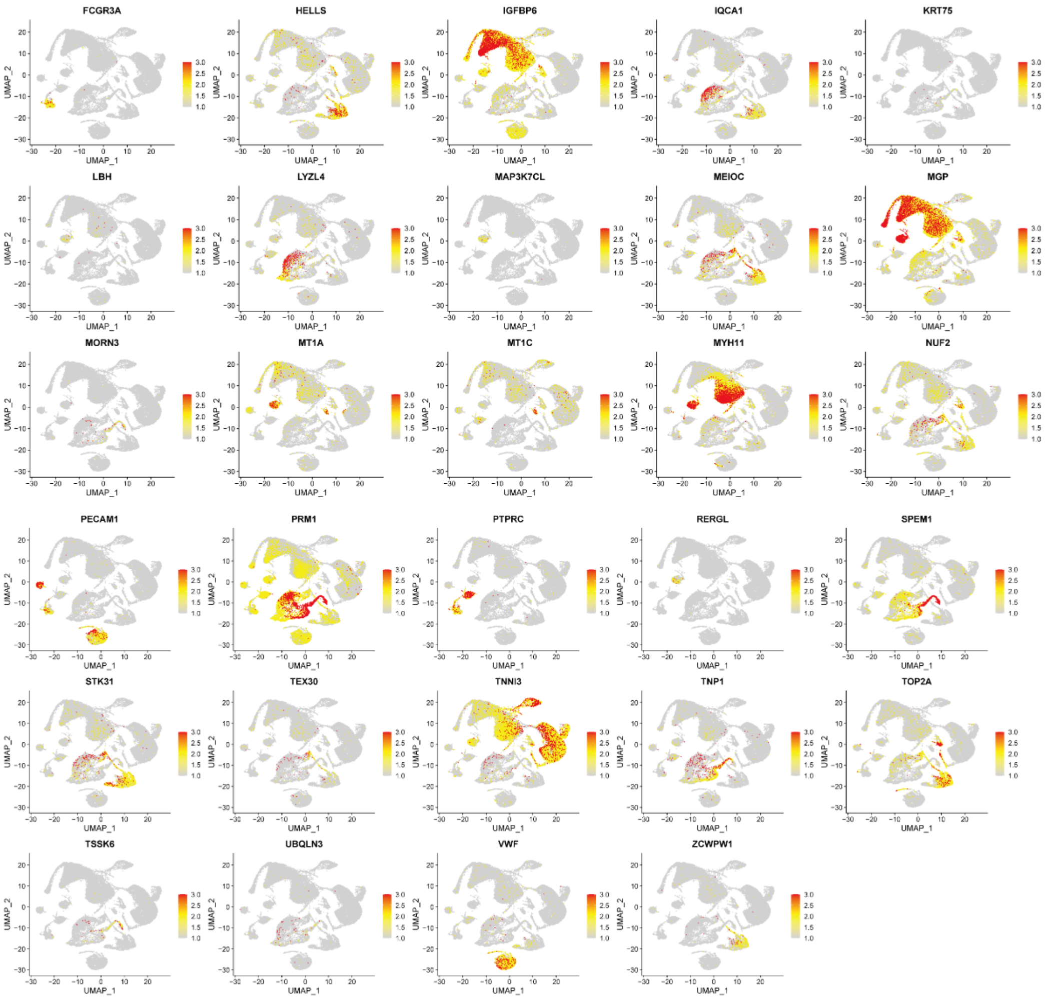

Figure S1. The expression patterns of marker genes projected on the UMAP plot. Red indicates high expression and gray indicates low or no expression.
